# Effects of the depth of the acetabular component during simulated acetabulum reaming in total hip arthroplasty

**DOI:** 10.1038/s41598-021-89292-3

**Published:** 2021-05-10

**Authors:** Jianlin Zuo, Meng Xu, Xin Zhao, Xianyue Shen, Zhongli Gao, Jianlin Xiao

**Affiliations:** 1 Department of Orthopedics, China-Japan Union Hospital of Jilin University, 126 Xiantai Avenue, Changchun, Jilin China; 2 Department of Orthopedics, The Second Hospital of Jilin University, 218 Ziqiang Road, Changchun, Jilin China

**Keywords:** Medical research, Preclinical research

## Abstract

We aimed to evaluate whether there are differences in the rotation center, cup coverage, and biomechanical effects between conventional and anatomical technique. Computed tomography scans of 26 normal hips were used to simulate implantation of acetabular component. The hip rotation center and acetabular component coverage rate were calculated. Moreover, a finite element model of the hip joint was generated to simulate and evaluate the acetabular cup insertion. Micromotion and the peak stress distribution were used to quantify the biomechanical properties. The medial and superior shifts of the rotation center were 5.2 ± 1.8 mm and 1.6 ± 0.7 mm for the conventional reaming technique and 1.1 ± 1.5 mm and 0.8 ± 0.5 mm for anatomical technique, respectively. The acetabular component coverage rates for conventional reaming technique and anatomical technique were 86.8 ± 4% and 70.0 ± 7%, respectively. The micromotion of the cup with conventional reaming technique was greater than that with anatomical technique. The peak stress concentration was highest in the superior portion with conventional reaming technique, whereas with anatomical technique, there was no stress concentration. Paradoxically although the acetabular component coverage rate is larger with conventional reaming technique, anatomical technique provides less micromotion and stress concentration for initial cup stability. Thus, anatomical technique may be more suitable for acetabulum reaming during primary total hip arthroplasty.

## Introduction

Total hip arthroplasty (THA) is successfully used to treat a broad spectrum of hip disorders and is associated with excellent long-term clinical outcomes^1–3^. THA can restore the natural acetabular rotation center and recover the natural function of the hip^4,5^. However, with the conventional reaming technique, orthopedic surgeons ream the acetabulum medial to the floor until a suitable component size for implantation is achieved, which results in medial and superior displacement of the rotation center, so as to obtain the approximate normal acetabular offset and the center of rotation of the hip. With the anatomical technique, the acetabulum is reamed peripherally and referenced from the rim, thus avoiding major displacement of the rotation center, especially in patients who have a thick pre-operative acetabular floor^6,7^.

The natural rotation center plays a key role in maintaining muscle function^8^ and in survival of the implant in THA^9^. Bicanic et al.^10^ reported that a 1% increase in hip load results in an approximately 10-mm displacement of the rotation center. Several studies have found that the hip anatomy is not fully restored following THA compared with the contralateral native hip, and 3-dimensional (3D) motion asymmetry of the hip and pelvis occurs in unilateral THA patients during gait^10–14^. However, the underlying cause of poor recovery of normal gait after THA remains unclear. Previous studies only focused on the differences in the anatomical rotation center between the conventional reaming technique and the anatomical technique^6,7^, and there are no reports in the literature regarding the differences in the cup coverage, biomechanical properties, and comprehensive analysis between these techniques. Generally speaking, insufficient cup coverage will increase the stress on the bone-cup interface, thereby increasing the probability of mechanical failure^15^. It is also considered to be one of the main unfavorable factors that cause acetabular loosening after THA. On the other hand, restoring the center of rotation is essential for successful THA. It can reduce the risk of bone or soft tissue impingement, thereby providing a better range of motion and reducing the risk of dislocation, and it can improve hip joint and abductor function^16^.

The finite element analysis method is an important way to investigate biomechanical properties in orthopedic studies, and we have previously demonstrated the effect of the Ganz ring on the acetabular bone defect during THA using this method^17,18^. The present clinical study aimed to compare the changes in the rotation center and cup coverage between the conventional reaming technique and anatomical technique using a 3D reconstruction method and to investigate differences in the micromotion and stress effects between the conventional technique and anatomical technique via finite element analysis.

## Materials and methods

### Subjects

The present study was approved by the Institutional Review Board, Ethics Committee of China-Japan Union Hospital (IRB No: 2020-NSFC-007). All patients provided a signed informed consent prior to undergoing the computer tomographic (CT) examination. All experiments were performed in accordance with relevant guidelines and regulations. This study included a total of 26 normal hips in 13 adult Chinese patients (7 men and 6 women), who underwent CT angiography for diagnosis of vascular diseases and whose data were recorded in the Digital Imaging and Communications in Medicine (DICOM) database. The average age was 43.1 years (range, 33–60 years). The average body mass index (BMI) was 25.3 ± 5 kg/m^2^. In order to avoid the interference of hip morphological variation on the reconstruction of rotation center, patients who had severe osteoarthritis in the hip or any deformities in the pelvis were excluded.

### CT scanning and 3D reconstruction

CT scans were performed using the same protocol with a Toshiba Aquilion CT scanner (120 kVp; 320 mA; 512 × 512 matrix; slice thickness, 0.5 mm). All patients were placed in a supine position on the scanner. The patient’s knees were taped to the scanner platform in an extended position with their patella facing towards the ceiling. CT scans were performed from the pelvis to the ankle joint in 0.5-mm slices. All CT slices were saved as DICOM files. The DICOM data were imported into Mimics 17.0 software (Materialise, Leuven, Belgium) for hip reconstruction.

### Hip rotation center and cup coverage measurement

**Figure 1:**
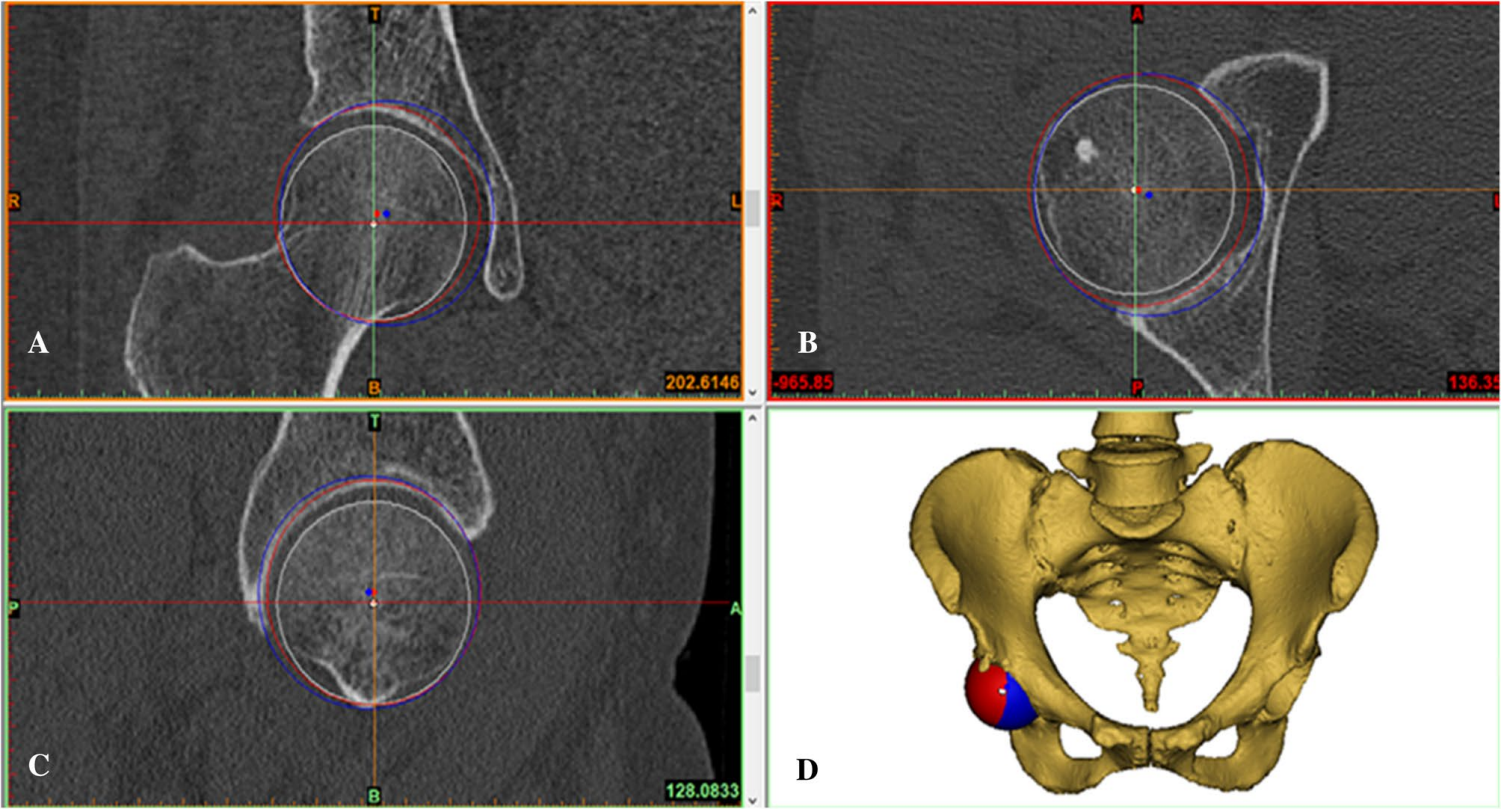
During the simulation of acetabular component implantation, the 3D, coronal, sagittal, and transverse views were presented simultaneously in Mimics software. Hip rotation center shift after application of the conventional reading technique and the anatomical technique in the coronal plane. The white point in the femoral head is the native hip rotation center; the blue point is the hip rotation center for conventional reaming technique; the red point is the hip rotation center for the anatomical technique. Medial shift and superior shift were determined by measuring the coronal and vertical distances from the blue and red points to the white point, respectively (**A**). (**B**–**D**). The axial plane (**B**), the sagittal plane (**C**), and the 3D model of the mimic implantation (**D**).

**Figure 2:**
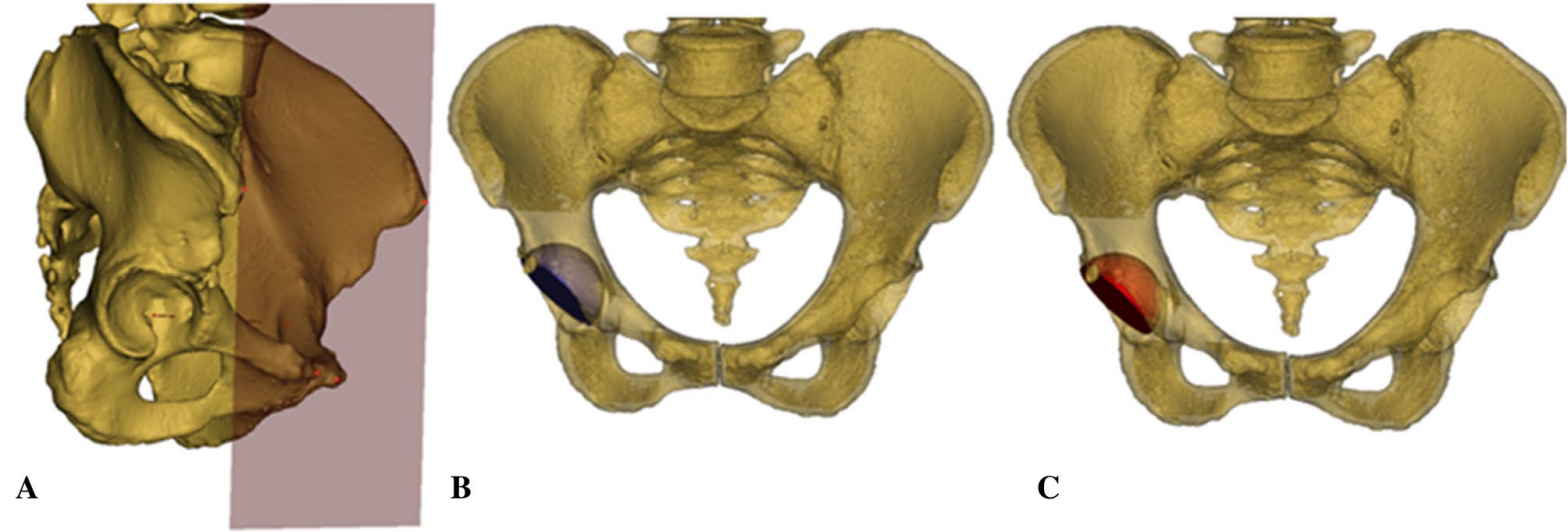
The 3D implantation simulations performed simultaneously in Mimics software. The pelvic position was standardized with reference to the anterior pelvic plane (**A**). The conventional reaming technique with cup inclination of 45° and anteversion of 15° (**B**). The anatomical technique with cup inclination of 45° and anteversion of 15° (**C**).

**Figure 3:**
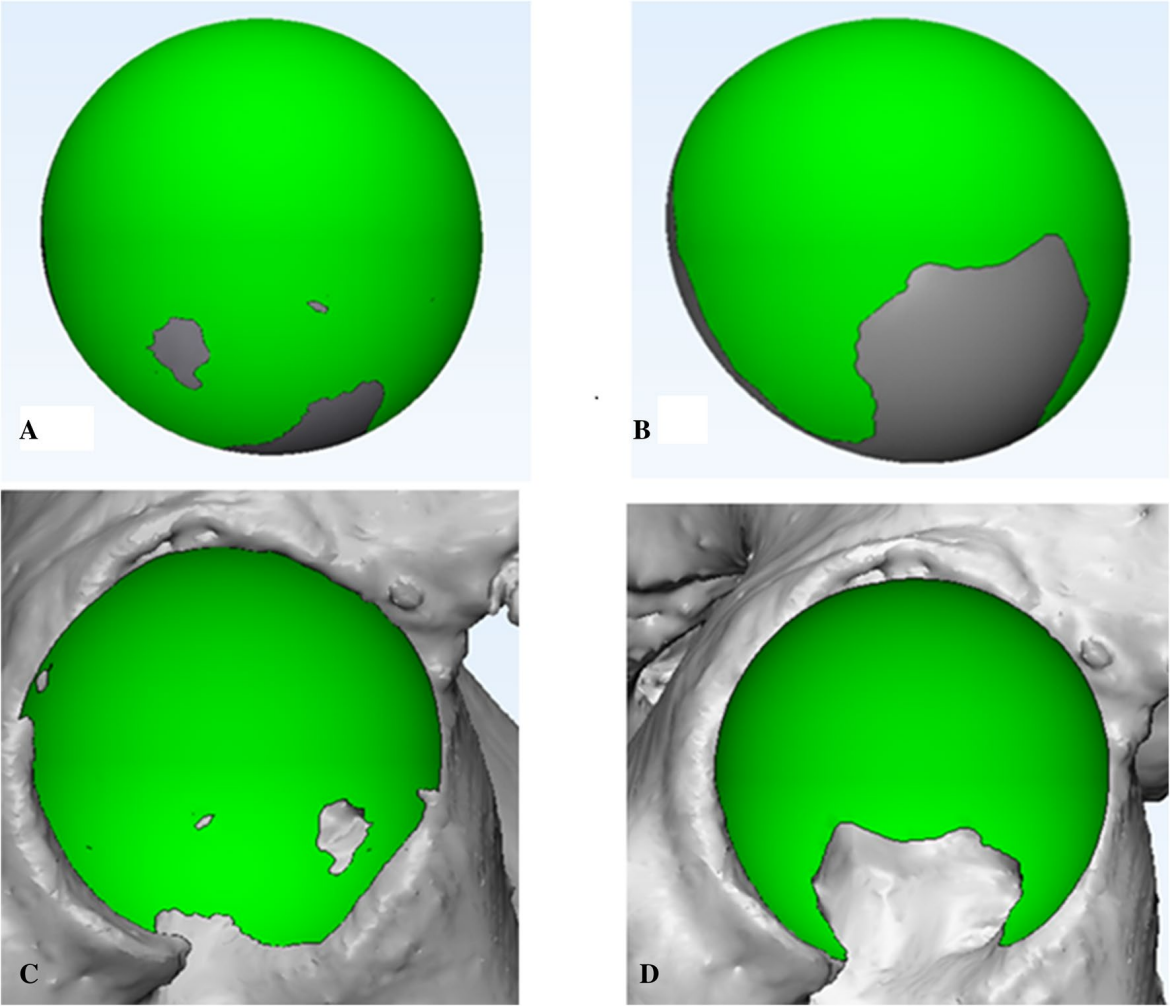
Interfaces between the cup component and the host acetabulum. (**A**, **B**). Cup coverage with the conventional reaming technique (**A**) and with the anatomical technique (**B**). (**C**, **D**). The interface on the acetabulum with the conventional reaming technique (**C**) and with the anatomical technique (**D**).

According to a study by Bonnin et al.^6^, the native hip rotation center is the femoral head center determined by the sphere fitting method. We used two spheres with different diameters to mimic the acetabular component implantation using two different methods: the conventional reaming technique and the anatomical technique (Fig. 1A–D). In the conventional reaming technique, the acetabular cup was in contact with the true floor of the acetabulum, but in the anatomical technique, the cup was positioned at the level of the subchondral bone. Then, the acetabular cup diameter with these two techniques was measured in the transverse plane. The medial and superior shifts of the rotation center were measured in the coronal plane in comparison with the native rotation center and verified in the axial and sagittal planes (Fig. 1A–C). Then, the pelvic position was standardized with reference to the anterior pelvic plane^19–21^ and the pubic tubercles (Fig. 2A). Thus, the coronal, axial, and sagittal images were reoriented according to the anterior pelvic plane and the line between the iliac spines. A plane passing through the center of the sphere was created to cut these spheres with different diameters to achieve a cup inclination of 45° and anteversion of 15° (Fig. 2B,C). The simulated acetabular replacement was performed by placing the component using two different reaming techniques. Component size was determined based on previous measurements of acetabular diameter. The component coverage ratio was then measured in the 3D environment. On the basis of the implantation simulation, an egg-shell cup with negligible thickness was developed to replace the implanted cup. Utilizing the simulation function of Mimics^21,22^, segmentations were performed according to the border between the covered and uncovered parts of the virtual cup (Fig. 3A,B). The coverage was calculated as the ratio of the covered area to the total surface area, which reflects the relative effective bone mass of the true acetabulum (Fig. 3C,D).

### Finite element analysis

In order to eliminate differences in patients' bone conditions, the sawbone (Pacific Research Laboratories, Inc., Vashon, WA) of the left pelvis was purchased and the hip geometry data were obtained by CT with a slice thickness of 0.6 mm. Then, hip geometry data was used to develop 3D finite element sawbone model using Mimics 17.0 software. The cortical bone profile and the boundary between the cortical and the trabecular bone were determined by the thresholding method and visual inspection when necessary. In the present study, two THA models (Fig. 2B,C) were developed. In the conventional reaming technique, the diameter of the mimic acetabular cup was 60 mm. In the anatomical technique, the diameter of the mimic acetabular cup was 58 mm. To simplify the model calculation, a sphere was used to substitute the acetabular cup. The interface between the cup and the pelvic bone was treated as a nonlinear contact problem, with friction set at 0.88 as previously reported by Zhang et al.^23^.

For the 3D model of the conventional reaming technique, a total element number of 240,329 and a total node number of 49,217 were created. For the 3D model of the anatomical technique, a total element number of 242,587 and a total node number of 49,433 were created. The 3D tetra 4 element was used to model the cortical and cancellous bone. The material property values of each element are summarized in Table 1 based on data reported in the literature^17,18,23,24^.

**Table 1:** Element types and material properties reported in the literature^17,18,23,24^.

Material	Element type	Young’s modulus (*E*: MPa)	Poisson’s ratio (ν)
Cortical bone	Solid	17,000	0.30
Trabecular bone	Solid	100	0.20
Prothesis	Solid	110,000	0.30
Surface	Contact	Friction coefficient	0.88

**Figure 4:**
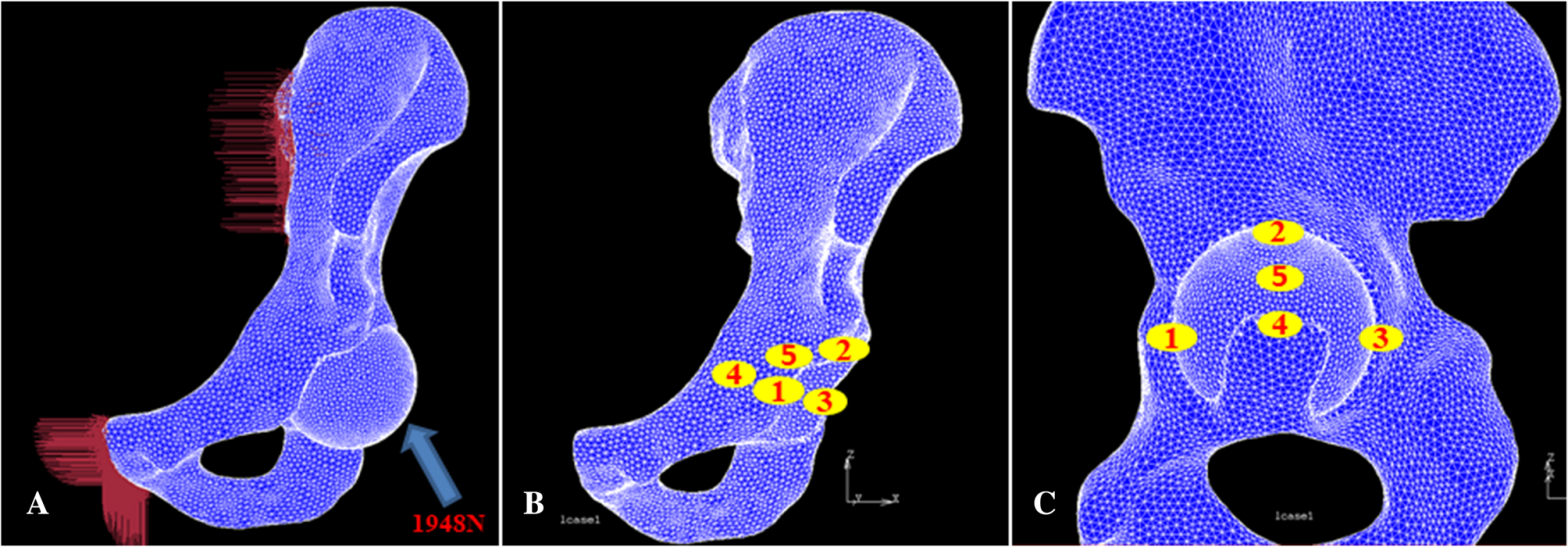
Finite element analysis models of the sawbone were developed and analyzed using MSC.Marc/Mentat2005r3 software. (**A**) Illustration of the measurement points for micromotion between the interface of the cup and host bone around the acetabulum. (**B**, **C**) The locations of 5 points from two different perspectives.

The loading condition used in this study was based on the report by Bergmann et al.^25^. In this model, the force magnitude and direction were normalized to 100% of the normal gait of THA patients, and the peak load was applied to the center of the head of each model (1948 N) (Fig. 4A). With regard to boundary conditions, the areas corresponding to the sacroiliac joint and pubic symphysis of the pelvic bone were considered completely restrained.

The models were developed and analyzed using MSC.Marc/Mentat2005r3 software (MSC Software Corp., Santa Ana, CA). For nonlinear analysis, force control was used for the numerical procedure. For an iterative method, the Newton–Raphson method was used with the load applied in 20 steps by an incremental loading method. The fixation strength of the cup in each model was evaluated based on the relative micromotion at the interface between the titanium acetabular cup and pelvic bone following load application at five points around the acetabulum. The five points we selected were the anterior and posterior column of the acetabulum (points 1 and 3), the superior portion of the acetabulum (point 2), the bottom of the acetabulum (point 4) and the midpoint of the superior and bottom of the acetabulum (point 5) (Fig. 4B,C).

### Statistical analysis

The mean and standard deviation were calculated for continuous variables (rotation center shift and cup coverage rate). Micromotion between the interfaces around the acetabulum was calculated. To assess if the effects of the two ream techniques on acetabular component motions were statistically different, paired *t*-tests were performed to determine the displacement of the hip rotation center and acetabular component coverage. All statistical analyses were conducted using SPSS version 21.0 software (SPSS, Chicago, IL, USA). *p* < 0.05 was considered statistically significant.

## Results

Table 2 summarizes the results of the hip rotation center shift in comparison with the native rotation center with use of the conventional reaming technique versus the anatomical technique. For the conventional reaming technique, the rotation center was medially shifted by 5.2 ± 1.8 mm and superiorly shifted by 1.6 ± 0.7 mm. For the anatomical technique, the rotation center was medially shifted by 1.1 ± 1.5 mm and superiorly shifted by 0.8 ± 0.5 mm. The mean shift values differed significantly between the two techniques (*p* < 0.001).

**Table 2:** Rotation center shift and cup coverage rate with different techniques.

	Anatomical reaming technique	Conventional reaming technique	*p*
Medial shift	1.1 ± 1.5 mm	5.2 ± 1.8 mm	< 0.001
Superior shift	0.8 ± 0.5 mm	1.6 ± 0.7 mm	< 0.001
Cup coverage rate	70.0 ± 7%	86.8 ± 4%	< 0.001

The acetabular component coverage rate was 86.8 ± 4% for the conventional reaming technique, which was significantly greater than that (70 ± 7%) for the anatomical technique (Table 2).

The micromotion of the cup was greater with the conventional reaming technique than with the anatomical technique (Table 3). With both techniques, the minimal micromotion occurred in the superior part of the acetabulum (Point 2), and the largest micromotion occurred in the bottom of the acetabulum (Point 4), which was 46 μm in the conventional reaming technique and 37 μm in the anatomical technique (Table 3).

**Table 3:** Micromotion between the interfaces around the acetabulum.

Point	Conventional reaming technique (μm)	Anatomical reaming technique (μm)
1	28	23
2	12.8	3.6
3	39	35
4	46	37
5	22	7

**Figure 5:**
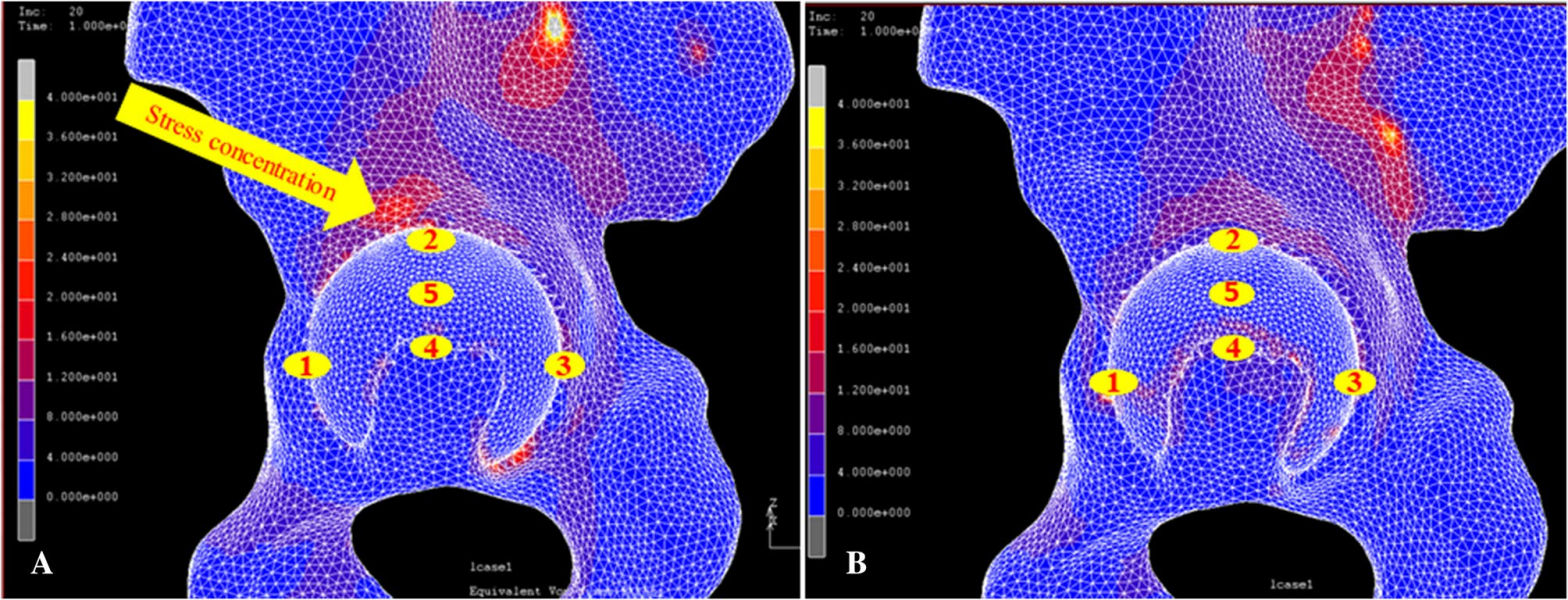
Illustration of the stress distribution and micromotion with the conventional reaming technique (**A**) and with the anatomical technique (**B**). The maxim micromotion (46 μm) was in the bottom of the acetabulum (Point 4) with the conventional reaming technique, and the stress concentration was in the superior portion of the acetabulum (yellow arrow) (**A**). The maximum micromotion (37 μm) was in the bottom of the acetabulum (Point 4) with the anatomical technique. There was no stress concentration in the superior portion of the acetabulum, and the stress was similar in the bottom and other areas (**B**).

The peak stress distribution was the highest in the superior portion with the standard reaming technique, whereas with the anatomical technique, no peak stress was found, which means that there was no stress concentration on the bone-cup interface. In addition, the stress in the bottom of the acetabulum was larger with the anatomical technique than with the conventional reaming technique (Fig. 5).

## Discussion

This study revealed two important findings. First, the hip rotation center following THA was influenced by the different reaming techniques, and the anatomical technique resulted in better biomechanical properties for initial cup stability did than the conventional reaming technique. Second, the cup coverage achieved with the conventional reaming technique was larger than that with the anatomical technique (86.8 ± 4% *vs* 70.0 ± 7%, respectively), and the hip rotation center shift was also larger with the conventional reaming technique (medial shift: 5.2 ± 1.8 mm *vs* 1.1 ± 1.5 mm; superior shift: 1.6 ± 0.7 mm *vs* 0.8 ± 0.5 mm, respectively). A large rotation center shift may decrease the offset widely compared with the native rotation center. This large rotation center shift should not be ignored by surgeons.

There is a consensus on the importance of the restoration of the native offset in THA^6,7,26,27^. Previous studies have shown that THA is associated with a higher incidence of loosening with medialization of the axis of rotation of the acetabular cup^28,29^. Karachalios et al.^30^ reported that displacement of the hip rotation center by ≥ 2 mm was strongly correlated with radiographic signs of loosening in 95 patients with THA within the 12- to18-year radiographic follow-ups. A similar result was observed in 83 juvenile patients with rheumatoid arthritis who received cemented THA, showing that acetabular migration in placed components by > 5 mm superiorly or medially may lead to progressive radiolucencies^31^. Using a computer model, Kurtz et al.^32^ reported that the hip rotation center shift has a greater effect on the range of movement before impingement than equivalent changes in femoral offset and femoral height. Therefore, some investigators have suggested that the anatomical technique should be used to prepare the acetabulum before cup component implantation^6,7^. Bonnin and colleagues^6^ measured CT data for 100 hips and found that the medial, superior, and posterior shifts of the hip rotation center were larger with the conventional reaming technique than with the anatomical technique. They suggest that surgeons should ream the acetabulum conservatively and place the acetabular cup anatomically in order to restore the native hip biomechanics. We support their opinion regarding conservative reaming of the acetabulum during primary THA. In addition, the findings of the present study show some additional advantages of this approach. First, we calculated the cup coverage, which is regarded as an indicator of the initial stability of the cup component. Our findings confirm that despite the fact that cup coverage is worse with the anatomical technique (70.0 ± 7%), there were unexpected biomechanical advantages. However, this theory may not be able to explain some acetabular morphological changes in some diseases such as DDH and acetabular bone defects, which had a cup coverage of less than 70%. We recommend that the surgeons should be encouraged to ream to the true floor so as to maximize compromised cover. Second, we performed a finite element analysis of the two different techniques and found that the micromotion of the acetabulum and stress distribution were greater with the conventional reaming technique than with the anatomical technique (Table 3). Our findings support the use of the anatomical technique for acetabulum reaming during THA. These results suggest that the high cup coverage rate does not always guarantee better initial cup stability.

Meermans et al.^7^ compared changes in the hip rotation center in 100 THA patients who were treated with the conventional reaming technique or the anatomical technique and found that the conventional reaming technique resulted in much greater medial and superior shifts of the hip rotation center than did the anatomical technique. Moreover, such shifts cannot be compensated by using a high offset stem. In addition, Charnley^33^ confirmed that improving the hip joint reactive force by increasing femoral offset sacrificed the range of movement. According to the biomechanical results, the anatomical technique exhibited a better stress distribution and smaller micromotion, as well as lower cup coverage rate compared with the conventional reaming technique. Most importantly, the stress distribution in the rim was similar to that in the bottom of the acetabulum with the anatomical technique (Fig. 5B). This may be because the subchondral bone, which supports the cup sturdily, was preserved with the anatomical technique, whereas with the conventional reaming technique, most of the subchondral bone was reamed, and the remaining cancellous bone had insufficient strength to support the cup. The difference in the preservation of subchondral bone may be responsible, at least in part, for the differences in micromotion and stress between the two techniques. Therefore, when we reamed the acetabulum, the cup coverage was not the only target we achieved. Preserving acetabular rim integrity and intact subchondral bone of the acetabulum was more important for initial cup stability.

## Limitations

There are several limitations in our study. First, the sample size of our study was relatively small (n = 26). Second, we only used the standard sawbone hip model for biomechanical analysis, although finite element analysis using the sawbone hip model is very commonly used in the literature^17,18^. However, this method cannot reflect the individual properties of each hip. Third, we simplified the cup as a sphere when we performed finite element analysis. This may affect the result, although we believe that this effect is very small. Finally, although our previous clinical study^34^ has confirmed that anatomical technique can achieve accurate reconstruction of the rotation center, this study reported the imaging and biomechanical findings, without clinical results to verify. Further clinical studies are required to confirm the findings of this study in the future.

## Conclusions

In summary, although the cup coverage with the anatomical technique was lower than that with the conventional technique, the anatomical technique was associated with better initial cup stability. In addition, the anatomical technique produced favorable changes in the acetabular rotation center, micromotion, stress distribution and the preservation of bone stock. However, further clinical studies are required to confirm whether the anatomical technique can result in good clinical outcomes.
